# The Utilization of Rehabilitation in Patients with Hemophilia A in Taiwan: A Nationwide Population-Based Study

**DOI:** 10.1371/journal.pone.0164009

**Published:** 2016-09-30

**Authors:** Chien-Min Chen, Yao-Hsu Yang, Chia-Hao Chang, Chih-Cheng Chen, Pau-Chung Chen

**Affiliations:** 1 Department of Physical Medicine and Rehabilitation, Chang Gung Memorial Hospital, Chiayi, Taiwan; 2 School of Medicine, College of Medicine, Chang Gung University, Taoyuan, Taiwan; 3 Hemophilia and Thrombosis Treatment Center, Chang Gung Memorial Hospital, Chiayi, Taiwan; 4 Department of Traditional Chinese Medicine, Chang Gung Memorial Hospital, Chiayi, Taiwan; 5 Institute of Occupational Medicine and Industrial Hygiene, National Taiwan University College of Public Health, Taipei, Taiwan; 6 Center of Excellence for Chang Gung Research Datalink, Chang Gung Memorial Hospital, Chiayi, Taiwan; 7 School of Traditional Chinese Medicine, College of Medicine, Chang Gung University, Taoyuan, Taiwan; 8 Department of Nursing, Chang Gung University of Science and Technology, Chiayi Campus, Chiayi, Taiwan; 9 Division of Hematology and Oncology, Department of Medicine, Chang Gung Memorial Hospital, Chiayi, Taiwan; 10 Department of Environmental and Occupational Medicine, National Taiwan University Hospital and National Taiwan University College of Medicine, Taipei, Taiwan; University of Hawai'i at Manoa, UNITED STATES

## Abstract

**Introduction:**

Rehabilitation plays an important role in the physical health of patients with hemophilia. However, comprehensive information regarding the utilization of rehabilitation for such patients remains scarce.

**Aim:**

This population-based study aimed to examine the characteristics, trends, and most important factors affecting rehabilitation usage in patients with hemophilia A using a nationwide database in Taiwan.

**Methods:**

Data from 777 patients with hemophilia A who were registered in the National Health Insurance Research Database between 1998 and 2008 were analyzed using SAS 9.0.

**Results:**

Musculoskeletal or nervous system-related surgical procedures and clotting factor VIII concentrate costs were identified as factors affecting rehabilitation usage; musculoskeletal or nervous system-related surgical procedures (odds ratio = 3.788; P < 0.001) were the most important predictor of whether a patient with hemophilia A would use rehabilitation services. Joint disorders, arthropathies, bone and cartilage disorders, intracranial hemorrhage, and brain trauma were common diagnoses during rehabilitation use. The costs of physical therapy (physiotherapy) comprised the majority (71.2%) of rehabilitation therapy categories. Increasingly, rehabilitation therapy was performed at physician clinics. The total rehabilitation costs were <0.1% of the total annual medical costs.

**Conclusion:**

Musculoskeletal or nervous system-related surgical procedures and increased use of clotting factor VIII concentrate affect the rehabilitation utilization of patients with hemophilia A the most. The findings in this study could help clinicians comprehensively understand the rehabilitation utilization of patients with hemophilia A.

## Introduction

The National Health Insurance (NHI) program was launched in Taiwan in 1995. This universal program covers all insured individuals and has enrolled more than 99% of all Taiwanese citizens and legal residents, with premiums generally ranging from 2% to 5% of the total household income [1]. The Taiwanese government, employers, and employees contribute different proportions of these premiums. More than 90% of all medical services hold contracts with the NHI Bureau. Insured individuals are free to select any NHI-contracted medical services of their choice.

Before the NHI programs were implemented, patients with hemophilia A received insufficient treatment with clotting factors. Since its initiation, the NHI has classified hemophilia as a catastrophic illness. This classification exempts patients with hemophilia from copayment for clinic visits or hospitalization associated with hemophilia-associated diseases or comorbidities.

Although research regarding the treatment of hemophilia has progressed to cell [2] and gene therapy [3, 4], patients with hemophilia still face clinical problems. Repeated episodes of hemarthrosis can cause joint pain and limit the range of motion [5]. Following joint procedures, in-hospital rehabilitation helps to restore this range of motion [6]. Additionally, evidence suggests that intensive rehabilitation could reduce joint pain [7, 8] and improve muscle circumference [8] in patients with hemophilia. Physical therapy (physiotherapy) may be beneficial for chronic hematomata and pseudotumor management in patients with hemophilia [9]. For patients with hemophilia and neurological sequelae after intracranial hemorrhage, rehabilitation undoubtedly plays an important role in the recovery of musculoskeletal functions [10]. Additionally, rehabilitation provides mental benefits. For young patients with hemophilia, educational physical therapy interventions can effectively improve family functioning and parents’ perceptions of stress [11].

Two previous studies [12, 13] mentioned rehabilitation cost in studies of patients with hemophilia from a nationwide Taiwanese database. However, rehabilitation was not the main research focus and was not discussed clearly in either article. Comprehensive information regarding rehabilitation service use among patients with hemophilia remains scarce. As patients with hemophilia A comprise the majority of hemophilic cases [14], we used a nationwide database to conduct a retrospective longitudinal study of patients with hemophilia A and examined the characteristics, trends, and most important factors affecting rehabilitation usage at outpatient and inpatient medical services.

## Materials and Methods

### Database

In Taiwan, the National Health Insurance Research Database (NHIRD) comprises de-identified personal data available for research purposes. This database includes important information such as beneficiaries, International Classification of Diseases, 9^th^ edition, Clinical Modification (ICD-9-CM) diagnostic codes and procedure codes, catastrophic illness and medical service registries, prescription orders, details, and expenditures at contracted pharmacies, and inpatient and outpatient claims and copayments. The NHIRD is among the largest administrative health care databases worldwide, and related studies have increased rapidly in both quality and quantity [15] since the first study was published in 2000.

### Identifying patients with hemophilia A

**Fig 1:**
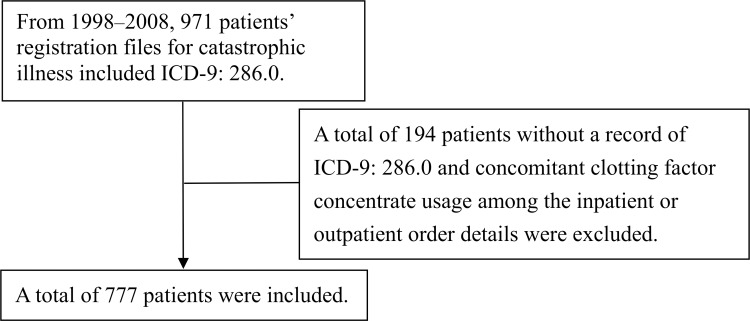
Patient selection flow chart.

The study protocol was approved by the Institutional Review Board for Human Studies at Chang Gung Memorial Hospital (approval number: 103-6124C). We included 777 patients registered for catastrophic illness between 1998 and 2008 whose medical records listed ICD-9: 286.0 and concomitant clotting factor concentrate (CFC) usage (Fig 1). CFCs included clotting factor VIII concentrates (sourced from human plasma or recombinant) and bypass agents (activated prothrombin complex concentrate or activated recombinant factor VII). Rehabilitation users were defined as patients receiving either inpatient or outpatient rehabilitation therapy. Rehabilitation non-users were defined those who received no rehabilitation therapy.

### Potential factors affecting rehabilitation usage

In previous articles, factors such as gender [12, 16], age [12], insured amount [12], urbanization [12], surgical procedures [12, 13], clotting factor VIII concentrate costs [17], and inhibitors (antibodies against factor VIII) status [13, 18] were suggested to affect medical resource utilization among patients with hemophilia A. These factors were available from NHIRD and were selected as potential factors affecting rehabilitation usage in this study.

The monthly insured amount was divided as < United States dollar (USD) 528 (New Taiwan dollar (NTD) 15840) and ≥USD 528 (NTD 15840) (conversion rate of NTD:USD = 30:1). Values of NTD 15840 represented monthly salaries at tier 1 according to the 38-tier insured amount designed by the NHI.

Places of residence (township level) for each year from 1998 to 2008 were estimated according to Lin [19], who combined insurance classifications, locations of hospital visits for respiratory tract infection, and insurance registrations. Urbanization was defined according to Liu [20], who stratified all 359 townships in Taiwan into 7 levels (1, most urbanized; 7, least urbanized) based on population density (people/km^2^), the proportions of people with college or higher educational levels, those older than 65 years, agriculture workers, and the number of physicians per 100,000 people according to Taiwan census data from the year 2000. This classification method, which has been used in other studies, provides the most accurate urbanization levels of all 359 townships in Taiwan [21, 22].

Because musculoskeletal and nervous system-related procedures are frequently associated with rehabilitation utilization, the procedures involving ICD-9-CM procedure codes 01–059 (nervous system) and 76–8499 (musculoskeletal system) were recorded.

The total costs for clotting factor VIII (including both human plasma and recombinant sources) were also recorded. Inhibitor status was considered the total cost of bypass agents, including activated prothrombin complex concentrate (FEIBA, Baxter AG, Vienna, Austria) and activated recombinant factor VII (NovoSeven, Novo Nordisk A/S, DK-2820 Gentofte, Denmark).

### Characteristics and trends of rehabilitation usage

In Taiwan, rehabilitation therapy is categorized as physical, occupational, or speech/swallowing therapy. The most common diagnosis (ICD-9-CM) associated with inpatient and outpatient rehabilitation prescriptions, the total costs in each rehabilitation therapy category and the number of rehabilitation users in terms of the number of rehabilitation therapy sessions received in both inpatient and outpatient services during 1998–2008, and the total number of rehabilitation therapy sessions at different medical institution levels were recorded. The total annual costs of inpatient and outpatient rehabilitation from 1998 to 2008 and the annual percentages relative to total medical costs were also surveyed. Recorded costs included all claims and copayments both with and without the catastrophic illness card used at NHI-contracted medical institutions. The number of patients who visited the same medical institution for outpatient rehabilitation and CFC prescriptions was also surveyed.

### Statistical methods

SAS 9.0 for Windows (SAS Institute, Cary, North Canolina, USA) was used for data management and analysis. For factors affecting rehabilitation usage from 1998 to 2008, continuous and categorical variables were analyzed with the independent *t* test and chi-square test, respectively. Variables were also used for the analyses with Pearson’s and Spearman’s correlation to check the collinearity. A logistic regression analysis was used to identify the most important factors for rehabilitation usage. A P-value <0.05 was considered statistically significant.

## Results

Table 1 lists the characteristics of variables affecting rehabilitation usage in patients with hemophilia A from 1998 to 2008. Table 2 lists the results of a logistic regression analysis of the most important factors affecting rehabilitation usage in patients with hemophilia A. Rehabilitation usage was significantly more frequent among patients who underwent musculoskeletal or nervous system surgeries than among those who did not (odds ratio = 3.788; P < 0.001). A patient with a clotting factor VIII concentrate cost of USD 10,000 had an odds ratio for rehabilitation usage of 1.005 (P = 0.002).

**Table 1:** Individual characteristics according to variables in patients with hemophilia A from 1998 to 2008.

Variables	Rehabilitation users (*n* = 321)	Rehabilitation non-users (*n* = 456)	P
Gender			0.296
Male	311 (96.9%)	435 (95.4%)	
Female	10 (3.1%)	21 (4.6%)	
Mean age (years)			0.002
< 20	109 (34.0%)	215 (47.2%)	
20 to < 36	108 (33.6%)	137 (30.0%)	
36 to < 51	71 (22.1%)	71 (15.6%)	
≥ 51	33 (10.3%)	33 (7.2%)	
Mean insured amount per month			<0.001
< USD 528	183 (57.0%)	320 (70.2%)	
≥ USD 528	138 (43.0%)	136 (29.8%)	
Urbanization level*			0.266
1 (most urbanized)	96 (29.9%)	123 (27.0%)	
2	108 (33.6%)	133 (29.2%)	
3	52 (16.2%)	102 (22.4%)	
4	41 (12.8%)	66 (14.4%)	
5	7 (2.2%)	6 (1.3%)	
6	10 (3.1%)	19 (4.2%)	
7 (least urbanized)	7 (2.2%)	7 (1.5%)	
Musculoskeletal or neurological surgical procedures			<0.001
Yes	141 (43.9%)	67 (14.7%)	
No	180 (56.1%)	389 (85.3%)	
Total costs (USD) for clotting factor VIII concentrates	545,762 ± 602,351	341,582 ± 445,437	<0.001
Total costs (USD) for bypass agents	53,641 ± 273,364	380 ± 5,622	<0.001
Mean cost (USD) for rehabilitation	843 (3–38402)	0	

USD, United States dollar.

*The urbanization level of each patient was determined according to the median value of urbanization level in the database from 1998 to 2008.

**Table 2:** Logistic regression analysis of factors that might affect rehabilitation usage in patients with hemophilia A.

Variables	Odds ratio	95% CI Lower	95% CI Upper	P
Gender				
Male	1.605	0.679	3.794	0.281
Female (reference group)	1.000			
Mean age				
< 20	0.676	0.355	1.286	0.233
20 to < 36	0.658	0.360	1.203	0.174
36 to < 51	0.844	0.445	1.601	0.604
≥ 51 (reference group)	1.000			
Mean insured amount per month				
< USD 528	0.741	0.482	1.141	0.174
≥ USD 528 (reference group)	1.000			
Urbanization level				
1 (reference group)	1.000			
2	1.143	0.761	1.717	0.520
3	0.647	0.404	1.037	0.070
4	0.937	0.554	1.584	0.807
5	1.478	0.432	5.065	0.534
6	0.774	0.325	1.847	0.564
7 (least urbanized)	1.127	0.339	3.745	0.845
Musculoskeletal or neurological surgical procedures				
Yes	3.788	2.629	5.460	<0.001
No (reference group)	1.000			
Costs for clotting factor VIII concentrates (per USD 10,000)	1.005	1.002	1.009	0.002
Costs for bypass agents (per USD 10,000)	1.142	0.967	1.347	0.117

USD, United States dollar; CI, confidence interval.

**Fig 2:**
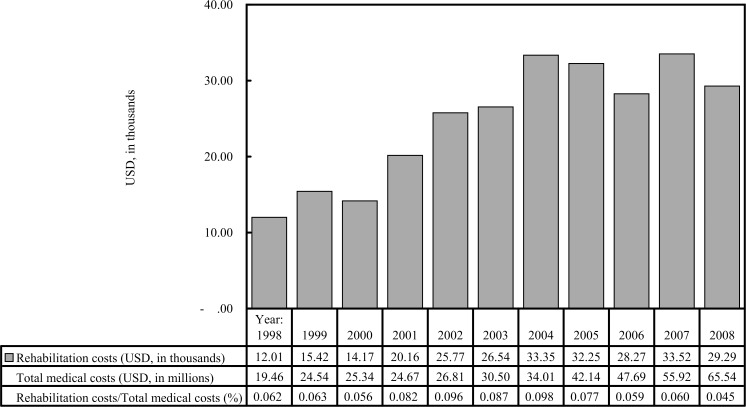
Annual rehabilitation costs and total medical costs in patients with hemophilia A from 1998 to 2008.

Joint disorders, arthropathies, bone and cartilage disorders, intracranial hemorrhage, and brain trauma were common diagnoses among the top 20 ICD-9-CM codes associated with rehabilitation prescriptions of outpatients and inpatients with hemophilia A (shown in S1 and S2 Tables). The total costs for physical, occupational, or speech/swallowing therapy among patients with hemophilia A during the study period are USD 184,279.8 (71.2%), 52,743.5 (20.4%), and 21,671.7 (8.4%) individually. Fig 2 lists the total annual costs for rehabilitation from 1998 to 2008 and the percentages relative to total medical costs. The total number of rehabilitation therapy sessions at all medical institutions increased from year 1998 (995 times) to year 2008 (2216 times). Persistently positive growth of the total number of rehabilitation year by year only occurred in the physician clinics (year 1998:166 times; year 2008: 761 times), but it did not occurred in medical centers, regional hospitals, and community hospitals. The annual rates for outpatients rehabilitation usage among all patients with hemophilia A from 1998 to 2008 ranged from 4.3% (29/672) in 1998 to 11.4% (89/747) in 2007 (Table 3), and 41.7% (134/321) of the outpatients rehabilitation users used outpatient rehabilitation services and clotting factor concentrates at the same hospitals. Table 3 shows the number of rehabilitation users in terms of the number of rehabilitation therapy sessions received.

**Table 3:** Distributions of total number of hemophilia A cases and number of rehabilitation users in terms of the number of rehabilitation therapy sessions received.

Year	1998	1999	2000	2001	2002	2003	2004	2005	2006	2007	2008
	Number of cases										
Patients with hemophilia A	672	687	691	701	708	719	729	737	742	747	749
Outpatient rehabilitation users	29	34	54	38	40	40	46	74	72	85	69
Total number of outpatient rehabilitation therapy sessions											
1	3	1	10	5	7	7	6	25	17	16	11
2–6	10	9	18	12	13	8	13	18	25	26	25
≥ 7	16	24	26	21	20	25	27	31	30	43	33
Inpatient rehabilitation users	9	6	6	4	9	6	8	12	8	16	11
Total number of inpatient rehabilitation therapy sessions											
1	1	2	0	0	3	1	0	3	2	1	1
2–6	5	2	3	2	5	2	5	6	4	10	7
≥ 7	3	2	3	2	1	3	3	3	2	5	3

## Discussion

In this study, musculoskeletal or nervous system surgery and clotting factor VIII concentrates costs were identified as factors predictive of rehabilitation usage in patients with hemophilia A. Musculoskeletal or nervous system surgeries comprised the most important factor affecting the use of rehabilitation among patients with hemophilia A. This suggests that a patient’s present need determines their decision regarding rehabilitation. Joint disorders, arthropathies, and bone and cartilage disorders were all listed among the top 20 diagnoses associated with rehabilitation. Preoperative physical therapy assessments could predict and establish the limits of postoperative rehabilitation for hemophilic patients aiming to receive elective orthopedic procedures [23]. The importance of postoperative rehabilitation, including early manual or mechanical mobilization to regain joint range of motion and muscle isometric contractions, light open kinetic chain exercises to restore muscle control, and ambulation training to establish walking function after joint arthroplasty, cannot be overemphasized [23]. Intracerebral hemorrhage and brain trauma were also not uncommon in hemophiliacs, whose bleeding tendencies place them at greater risk of hemarthrosis or soft tissue hemorrhage, particularly in easily damaged organs such as the brain. Intracerebral hemorrhage affects 3.5–4% of male newborns with hemophilia [24]. Intracerebral hemorrhage also occurs frequently after the neonatal period, affecting 3–10% of the hemophilia population, who are mainly treated on demand [24]. Although the mortality associated with intracranial hemorrhage has decreased in the modern treatment age [25], sequelae of spontaneous or trauma-induced intracranial hemorrhage, including limb weakness, speech disorders, and swallowing dysfunction, will require rehabilitation to promote recovery.

The costs associated with clotting factor VIII might result from the practice of clotting factor VIII use in patients with hemophilia. Some patients may use clotting factor VIII prophylactically to avoid bleeding during rehabilitation. Also, increased usage of clotting factor VIII might reflect more severe hemophilia, which is associated with greater destruction of the major joints. Patients with greater joint destruction may require more rehabilitation resources to maintain an appropriate musculoskeletal condition.

Our regression model did not identify urbanization level as a factor. Lin et al. [21] used the NHIRD to evaluate the association between urbanization and outpatient service utilization in Taiwan and found that differences between urbanization levels were a major contributing factor associated with the probability and frequency of outpatient utilization. However, in that study [21], the multivariate logistic regression analysis identified only urbanization levels 3, 4, and 6 (relative to level 1); not all levels were significantly associated with outpatient service utilization. In our regression model, no urbanization level was significantly associated with rehabilitation usage. We believe that the patients in our study had ready access to medical institutions providing rehabilitation, which likely explains why the urbanization level was not a factor.

In Taiwan, increasing numbers of physician clinics have been established to provide rehabilitation services. According to our data, although CFC prescriptions were all provided by hospital-level medical services, approximately 40% of the patients with hemophilia sought rehabilitation and CFC prescriptions at the same hospitals. In other words, patients chose to receive rehabilitation both at hospital-level medical services and at physician clinics providing rehabilitation therapy. Although increasing numbers of Hemophilia Treatment Centers, which provide a wide variety of multidisciplinary interventions, have been established at Taiwanese hospital-level medical institutions, it remains questionable whether these centers can induce patients to undergo rehabilitation at hospitals.

Physical therapy accounted for the majority of costs among the 3 rehabilitation therapy categories. Physical therapy not only played a role in the conditions of patients before and after musculoskeletal surgery [23], but also helped patients with chronic hemophilic arthropathy to avoid surgery. Muscular atrophy often accompanies hemophilic arthropathy. To maintain a muscle condition sufficient to protect against progressive joint degeneration, the joint range of motion must be maintained and the muscles should be strengthened [26, 27]. Physical therapy is usually required. Physical therapy exercise programs, which target flexibility, strength, proprioception, balance, and functional training, can help to manage recovery after hemarthrosis or muscle bleeding or can help to prevent recurrent bleeding episodes [28]. Additionally, physical therapy could assist with pain relief and enhanced functional ability in patients with hemophilia [29]. However, physical therapy for pain relief is thought to be underutilized in this population [30]. According to a previous report, only 11% and 16% of patients with hemophilia used physical therapy for acute and persistent pain management, respectively [30]. According to our data, rehabilitation may also be underutilized in Taiwan. The annual rates for outpatient rehabilitation usage among all patients with hemophilia A from 1998 to 2008 were no more than 11.4%. A physical therapist should be able to professionally and regularly assess the joint range of motion, muscle volume and strength, and gait, educate patients adequately and efficiently to perform exercises either at the clinic or at home, and help the patient to safely engage in sport to maintain good musculoskeletal function and fitness and avoid becoming overweight. Professional conduct and the provision of physical benefits to patients with hemophilia might provide motivation for rehabilitation usage and exercise. Furthermore, while providing rehabilitation to patients with hemophilia, certain therapeutic strategies could be implemented, such as group therapy [31], which is frequently used in rehabilitation therapy to enhance motivation, and personal health passports or self-monitoring systems [32], which could improve exercise adherence and provide feedback to therapists.

Although the rehabilitation costs have increased since 2004, these values have fluctuated without additional year-over-year increases. However, the proportion of rehabilitation costs among the total medical costs has decreased annually since 2005; this is likely due to the dramatic and rapid increase in the costs associated with CFC use, as these comprised the majority (>85%) of total medical costs among patients with hemophilia A in our study. However, rehabilitation costs, unlike CFC costs, did not exhibit persistent year-over-year growth since 2005. Since 2005, the government of Taiwan has allocated an independent budget for NHI-covered rare diseases (including hemophilia) to counteract deficiencies in medical expenditures for these diseases. If the annual costs for rare diseases exceed the initial independent budget, the overage would be diverted from other budgets. We believe that this change in policy had a stronger influence on CFC usage than on rehabilitation resource usage.

In our study, rehabilitation costs accounted for <0.1% of the total annual medical costs. To our knowledge, few articles have reported the cost of rehabilitation usage relative to the total medical costs for patients with hemophilia. In Belgium, Henrard et al. reported that the costs of physiotherapist visits (€2,024,912) accounted for 2.2% of the total direct costs for 1153 patients with hemophilia in 2011 [33]. Ballal et al. demonstrated that inpatient rehabilitation costs accounted for 0.12–0.84% of the total estimated costs for different knee-related procedures in hemophilic patients using high-titer inhibitors [34]. Rehabilitation costs may differ among countries. In Spain, the unit cost of rehabilitation consultation is €24.26 (NTD:€ = 33.8:1) [35]. In Sweden, the hourly cost of physical therapy is €114 [36]. In Canada and the USA, the unit costs for physical therapy assessments are 30.73 Canadian dollars (CAN) and USD 110.78, respectively (NTD:CAN = 24.9:1) [37]. Physical therapy costs are relatively lower in Taiwan than in the above-mentioned countries. NHI claims cover most rehabilitation therapy costs. Physical therapy claims range from NTD 95–600 (USD 3.17–20), depending on the medical institution level. A patient may have 6 (physical, occupational, or speech/swallowing) therapy sessions after visiting a physiatrist. Depending on the degree of physical therapy, some patients either have no copayment or only a copayment of NTD 50 (USD 1.67) before the second through sixth physical therapy sessions.

Our study shares the limitations common to retrospective database analyses. We were unable to obtain additional information regarding factors that influence rehabilitation usage. In addition, we were also unable to acquire the database after the year 2008 owing to the limited grant. Furthermore, the diagnoses were sourced from NHIRD according to ICD-9-CM codes. These diagnoses might have been less accurate than diagnoses collected prospectively according to standard procedures. Despite these limitations, our study provides a clear profile of rehabilitation usage among Taiwanese hemophiliacs.

## Conclusions

This is the first comprehensive study to describe nationwide rehabilitation utilization in patients with hemophilia A. Musculoskeletal or nervous system surgeries and clotting factor VIII concentrates costs were all factors predictive of rehabilitation usage. Joint disorders, arthropathies, bone and cartilage disorders, intracranial hemorrhage, and brain trauma were common diagnoses among those using rehabilitation services. Only nearly 40% of the patients, however, sought outpatient rehabilitation services and CFC prescriptions at the same hospitals. Rehabilitation therapy has been increasingly performed at physician clinics. It is essential to promote the use of rehabilitation resources to achieve better muscle and joint condition among this population. It is also imperative to enhance the professional abilities of therapists to care for hemophilic patients.

## Supporting Information

S1 Table Top 20 ICD-9-CM codes associated with rehabilitation prescriptions for outpatients with hemophilia A, in decreasing order. ICD-9-CM, The International Classification of Diseases, Ninth Revision, Clinical Modification.
(DOC)

S2 Table Top 20 ICD-9-CM codes associated with rehabilitation prescriptions for inpatients with hemophilia A, in decreasing order. ICD-9-CM, The International Classification of Diseases, Ninth Revision, Clinical Modification.
(DOC)
