# Supplementary material for: The Utilization of Rehabilitation in Patients with Hemophilia A in Taiwan: A Nationwide Population-Based Study
Source: PLoS One. 2016 Sep 30;11(9):e0164009. doi: 10.1371/journal.pone.0164009 (PMC5045205; doi:10.1371/journal.pone.0164009)
Supplement: S1 Table — ICD-9-CM, The International Classification of Diseases, Ninth Revision, Clinical Modification. (DOC) [file pone.0164009.s001.doc]

| ICD-9-CM code | Disease |
| --- | --- |
| 286 | Coagulation defects |
| 718 | Other derangement of joint |
| 315 | Specific delays in development |
| 716 | Other and unspecified arthropathies |
| 713 | Arthropathy associated with other disorders classified elsewhere |
| 431 | Intracerebral hemorrhage |
| 715 | Osteoarthrosis and allied disorders |
| 726 | Peripheral enthesopathies and allied syndromes |
| 851 | Cerebral laceration and contusion |
| 342 | Hemiplegia and hemiparesis |
| 314 | Hyperkinetic syndrome of childhood |
| 724 | Other and unspecified disorders of back |
| 733 | Other disorders of bone and cartilage |
| 719 | Other and unspecified disorder of joint |
| 729 | Other disorders of soft tissues |
| 784 | Symptoms involving head and neck |
| 343 | Infantile cerebral palsy |
| 845 | Sprains and strains of ankle and foot |
| 721 | Spondylosis and allied disorders |
| 299 | Psychoses with origin specific to childhood |
