# Supplementary material for: The Utilization of Rehabilitation in Patients with Hemophilia A in Taiwan: A Nationwide Population-Based Study
Source: PLoS One. 2016 Sep 30;11(9):e0164009. doi: 10.1371/journal.pone.0164009 (PMC5045205; doi:10.1371/journal.pone.0164009)
Supplement: S2 Table — ICD-9-CM, The International Classification of Diseases, Ninth Revision, Clinical Modification. (DOC) [file pone.0164009.s002.doc]

| ICD-9-CM code | Disease |
| --- | --- |
| 286 | Coagulation defects |
| 715 | Osteoarthrosis and allied disorders |
| 713 | Arthropathy associated with other disorders classified elsewhere |
| 070 | Viral hepatitis |
| 719 | Other and unspecified disorder of joint |
| 718 | Other derangement of joint |
| 852 | Subarachnoid, subdural and extradural hemorrhage, following injury |
| 348 | Other conditions of brain |
| 401 | Essential hypertension |
| V43 | Organ or tissue replaced by other means |
| 599 | Other disorders of urethra and urinary tract |
| 716 | Other and unspecified arthropathies |
| 730 | Osteomyelitis, periostitis and other infections involving bone |
| 733 | Other disorders of bone and cartilage |
| 767 | Birth trauma |
| 820 | Fracture of neck of femur |
| 342 | Hemiplegia and hemiparesis |
| 431 | Intracerebral hemorrhage |
| 728 | Disorders of muscle, ligament and fascia |
| 853 | Other and unspecified intracranial hemorrhage following injury |
